# Dissertatio de Preternaturali et raro Intestini Recti cum Vesica Urinaria coalitu et independente Ani defectu, Observationibus Anatomicis superstructa

**Published:** 1782

**Authors:** 


III. H. A. Wrisberg, M. D. & *Anatomes Professoris, Dissertatio de Preternaturali et raro Intestini Recti cum Vesica Urinaria coalitu et inde pendente Ani defectu, Observationibus Anatomicis superstructa. Cum figuris.* 4to. Gottingæ. 1779.

THIS dissertation, which has been announced in a former number of our Journal, seems to be entitled to a more particular notice, as containing a very accurate account of a curious anatomical fact, which is briefly as follows.

On the 17th of December, 1777, Mr. Kaufmann, surgeon at Gottingen, was desired to visit a child two days old, who was said to have no anus, but in other respects was apparently healthy and well formed. Mr. Kaufmann found this infant voiding at the mouth a frothy, bilious mucus, mucus, mixed with milk. He immediately requested the affiftance of our author, who advifed the making an artificial anus. An incifion was accordingly made in the centre of the fpace between the coccyx and fcrotum. The knife was carried flowly and cautioufly through a confiderable portion of fat, into the pelvis, but without fuccefs.

As it feemed impoffible to difcover the courfe of the inteftine, and there was danger of wounding the bladder, they agreed not to attempt any farther operation, but to leave the affair to nature. Soon after this, at the fecond vifit, they found the child's linen ftained with the meconium, a fmall quantity of which had been difcharged from the urethra. This circumftance led our author to imagine that the rectum communicated with the bladder.

The artificial anus being of no ufe, the lips of the wound were brought together by future. In order to promote the difcharge of the meconium, warm milk was injected into the urethra, but without the defired effect, The child languifhed till the 23d of December, when it died.

Upon diffection it was difcovered, that about the fecond vertebra of the facrum, the rectum had had deviated from its ordinary courſe, and paſſing tranſverſely towards the bladder, ſuddenly leſſened into a cone, which terminated at the poſterior part of the bladder, with which it communicated by a ſmall orifice that would with difficulty admit a pin's head.

Dr. Wriſberg obſerves, that this rectum, if it may be ſo called, ſeemed to be only a continuation of the colon or iliac plexus, and that the true rectum appeared to be altogether deficient: for the longitudinal bands, uſual in the colon, were diſtinctly continued in this new inteſtine, even to its extremity; whereas in other ſubjects they change into a broad thick muſcular coat, which belongs to the true rectum, and of which there was hardly any veſtige in the one here ſpoken of.

This ſingular termination of the rectum in the bladder was not the only extraordinary circumſtance obſerved in this ſubject. Two muſcles, viz. the *ſphincter* and *levator ani* were entirely wanting; and a third, the *accelerator urinæ*, or the *bulbo-cavernoſus*, as our author calls it in imitation of Winſlow, was very different from what it uſually is.

